# The association between ABO blood type and outcomes following sudden cardiac arrest: a multicenter observational study

**DOI:** 10.3389/fmed.2025.1525575

**Published:** 2025-04-08

**Authors:** Jiun-Wei Chen, Ren-Jie Tsai, Cheng-Yi Fan, Sih-Shiang Huang, Ching-Yu Chen, Chi-Hsin Chen, Jia-How Chang, Edward Pei-Chuan Huang, Wei-Tien Chang, Chih-Wei Sung

**Affiliations:** ^1^Department of Emergency Medicine, National Taiwan University Hospital Hsin-Chu Branch, Hsinchu, Taiwan; ^2^Institute of Molecular Medicine, National Tsing Hua University, Hsinchu, Taiwan; ^3^Graduate Institute of Clinical Medicine, College of Medicine, National Taiwan University, Taipei, Taiwan; ^4^Department of Emergency Medicine, National Taiwan University Hospital Yun-Lin Branch, Yunlin, Taiwan; ^5^Department of Emergency Medicine, College of Medicine, National Taiwan University, Taipei, Taiwan; ^6^Graduate Institute of Biomedical Informatics, College of Medical Science and Technology, Taipei Medical University, Taipei, Taiwan; ^7^Department of Emergency Medicine, National Taiwan University Hospital, Taipei, Taiwan

**Keywords:** ABO blood type, cardiac arrest, neurological outcome, sudden cardiac arrest, survival

## Abstract

**Background:**

ABO blood type has been associated with various disease outcomes, but its relationship with outcomes in patients with sudden cardiac arrest (SCA) remains unexplored.

**Methods:**

This was a retrospective analysis of adult out-of-hospital cardiac arrest patients with SCA treated at three major branches of the National Taiwan University Hospital between January 2016 and July 2023. The variables examined for their possible influence on the neurological and survival outcomes of patients with SCA were sociodemographic characteristics, pre-existing diseases, resuscitation events, and blood type. The results of a multivariable logistic regression were reported as adjusted odds ratios (aORs) and 95% confidence intervals (CIs). Neurological outcomes were determined by the Cerebral Performance Category (CPC) scale at hospital discharge.

**Results:**

No significant differences were found in the prevalence of each blood type between those who survived and those who did not or between those with good (CPC 1–2) or poor (CPC 3–5) neurological outcomes. There was no significant association between survival and blood type; however, patients with blood type AB had a higher probability of good neurological outcomes than those with blood type O (aOR: 1.98, 95% CI: 1.02–3.83, *p* = 0.042). A sensitivity analysis of the data from patients with aseptic etiologies also showed a significantly higher likelihood of good neurological outcomes among those with blood type AB (aOR: 2.21, 95% CI: 1.12–4.35, *p* = 0.023).

**Conclusion:**

ABO blood type is not associated with survival in patients with SCA, but blood type AB is associated with better neurological outcomes than type O.

## 1 Introduction

Despite advances in resuscitation and critical care medicine, there have been limited improvements in the outcomes of patients with sudden cardiac arrest (SCA). Only a small percentage of patients with SCA survive to hospital discharge, and even fewer have favorable neurological outcomes (1–3). While survival remains a fundamental measure of CPR success, neurological outcomes are increasingly acknowledged as a critical endpoint, as they more accurately reflect the long-term quality of life for both patients and their families (3). Numerous factors have been investigated for their potential effects on SCA outcomes (1, 4, 5). These include initial cardiac rhythm, time to defibrillation, whether bystander cardiopulmonary resuscitation (CPR) was performed, and whether the cardiac arrest was witnessed (4). The effects on outcomes of sociodemographic factors such as age, sex, body mass index (BMI), and socioeconomic status have also been investigated (6, 7). Notably, there has been relatively little research to date on the relationships between ABO blood types and disease outcomes.

Numerous factors, including structural heart disease, arrhythmic substrates, and metabolic disorders, are known risk factors for SCA (7, 8). ABO blood type has been implicated in the risk of venous thromboembolism, malignancies, and infectious complications, which may indirectly influence the incidence or outcomes of SCA (9–15). Specifically, blood type A has been associated with an increased risk of acute respiratory distress syndrome (ARDS) following both major trauma and severe sepsis (9, 10). ABO blood type has also been found to correlate with variations in susceptibility to SARS-COVID-19 and the severity of the infection (11, 12). Blood type O has been found to increase the risks of endometrial and pancreatic cancer (13, 14). The ABO type may also be associated with the risk of coagulopathy. Blood type O has been linked to lower plasma von Willebrand factor in healthy individuals (15). A meta-analysis by Urabe et al. has shown a higher incidence of venous thromboembolism (VTE) in those with non-O blood type than those with other blood types (16). These findings suggest the potential importance of considering ABO blood type in clinical assessments and treatment strategies for various medical conditions, including SCA (17, 18). However, whether ABO blood type significantly influences or correlates with the survival odds and neurological recovery of patients with SCA has not yet been explored.

We hypothesize that patients with SCA with certain blood types may have increased survival rates or better neurological outcomes. This study aims to investigate the association between ABO blood type and outcomes in patients with SCA. We hope to provide new insights that may inform future patient management strategies.

## 2 Materials and methods

### 2.1 Study design and ethics statement

This retrospective study analyzed data from the National Taiwan University Hospital, Hsinchu and Yunlin Branch Cardiac Arrest Research Database (NTUH-HYCARD). Access to the database was provided by the Departments of Emergency Medicine at the National Taiwan University Hospital (NTUH) and its branch hospitals. This included three hospitals across six campuses: the main NTUH (Taipei), the NTUH Hsinchu branch (comprising the Hsinchu, Biomedical Park, and Chu-Tung campuses), and the NTUH Yunlin branch (comprising the Huwei and Douliu campuses).

Ethical approval for this research was obtained from the Institutional Review Board (IRB) of the National Taiwan University Hospital (NTHU) (IRB code: 202401013RINC). The written informed consent requirement was waived because of the retrospective, observational design of the study. The study was conducted in accordance with the tenets of the 2013 revision of the Declaration of Helsinki.

### 2.2 Inclusion and exclusion criteria

**FIGURE 1:**
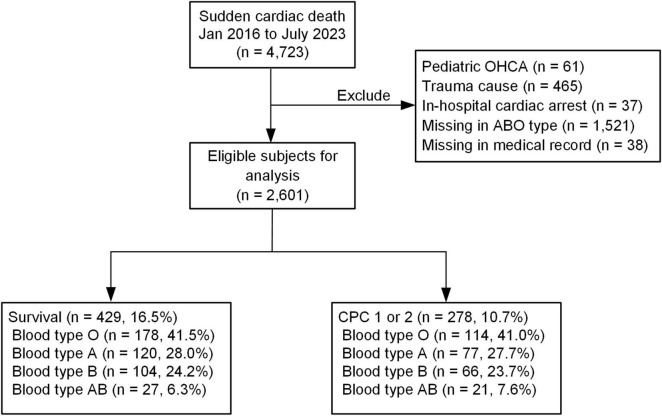
Patient enrollment flow: this figure depicts the patient screening and enrollment process. Initially, all patients were screened, and those who met the predefined exclusion criteria were excluded from the study.

Sudden cardiac arrest patients with out-of-hospital cardiac arrest (OHCA) were included in this study if emergency medical technicians attended the OHCA, they were aged 18 or over, they were brought to one of the hospitals included in this study for resuscitation, and the OHCA occurred between January 2016 and December 2023. Patients with trauma etiology for the SCA were excluded because the pathophysiology and management priorities of traumatic cardiac arrest differ significantly from those of medical (non-traumatic) cardiac arrests. Hemorrhagic shock and traumatic injuries dominated the outcome pathways in traumatic cardiac arrest, whereas medical OHCA was more often influenced by cardiac, respiratory, or metabolic etiologies. The other exclusion criteria included the occurrence of hospital transfer during or after resuscitation, blood type unknown or missing from the records, and data on outcome variables missing from the records. Patients with incomplete primary outcome data, meaning missing documentation for either survival to discharge or neurological outcome at discharge, were excluded. A flow chart of the patient recruitment process is provided in Figure 1.

### 2.3 Variables

The data were sourced from the NTUH-HYCARD and handled by a dedicated principal investigator and an independent team of researchers. The principal investigator held monthly meetings to ensure consistency in data collection. The team meticulously reviewed medical records and emergency medical services (EMS) dispatch reports to optimize data recovery and minimize potential biases. Data entry and organization was conducted using Research Electronic Data Capture (REDCap) (19, 20), a secure, web-based application for research data collection and collation (21).

Data collected from patient records were basic sociodemographic and clinical characteristics such as age, sex, and BMI; blood type (A, B, AB, or O); pre-existing medical conditions including hypertension, diabetes mellitus, hyperlipidemia, chronic kidney disease, and malignancies; resuscitation information, including whether cardiac arrest occurred in the presence of witnesses, whether CPR was performed by a bystander, ambulance response time, initial cardiac rhythm, whether defibrillation was performed, and low-flow time; and patient laboratory values, including blood cell count, C-reactive protein (CRP) level, lactic acid level, platelet count, and international normalized ratio (INR). The blood data were obtained on the hospital admission. The response time was defined as the duration between the time of the call to the emergency services and the time the dispatched EMS arrived at the scene (22). Initial rhythm was defined as the first cardiac rhythm detected when a monitor or defibrillator was applied after the cardiac arrest. Low-flow time was defined as the duration from EMS arrival at the scene to the return of spontaneous circulation.

### 2.4 Outcomes

The primary outcomes for this study were patient survival and patient neurological outcomes. The former was defined as survival status at hospital discharge. The latter was defined as patient Glasgow-Pittsburgh Cerebral Performance Category (CPC) scores at hospital discharge (23), which were evaluated by the attending critical care physician. CPC scores of 1 and 2 were classified as good neurological outcomes and 3–5 as poor neurological outcomes.

### 2.5 Statistical analysis

Categorical variables were expressed as numbers and percentages, and comparisons between these groups were performed using chi-squared tests. The normality of distributions was determined using the Kruskal–Wallis test. Normally distributed continuous variables were reported as mean ± standard deviation, and comparisons between outcome groups for these variables were conducted using student’s *t*-tests. Univariate analyses were performed, and variables with *p*-values < 0.1 were included in a multivariable logistic regression. We applied multivariable logistic regression model for both survival (binary) and neurological outcome (binary), controlling for sex, age, comorbidities (hypertension, diabetes mellitus, chronic kidney disease), witnessed arrest, bystander CPR, initial shockable rhythm, low-flow time, and laboratory values (as significant). The results of this regression were presented as adjusted odds ratios (aORs) and 95% confidence intervals (CIs). A subsequent sensitivity analysis was then performed to evaluate the robustness of the association between ABO blood type and SCA outcomes in different subsets of the study population. Two additional subset analyses (prespecified in our study protocol) were also conducted. The first compared the outcomes of patients of each blood type in a cohort whose SCAs had non-cardiac causes, and the second of patients in an asepsis cohort.

Statistical significance was determined using a two-tailed *p*-value threshold of < 0.05. All statistical analyses were performed using SPSS, v. 26 (IBM Corp., Armonk, NY, United States) and R software v. 4.4.1 (R Foundation for Statistical Computing, Vienna, Austria).

## 3 Results

### 3.1 Survival rates and neurological outcomes

From the hospitals included in this study, we identified 4,723 patients who suffered SCAs between January 2016 and July 2023. Of these, 2,122 patients were excluded. The reasons for exclusion were traumatic SCA etiology (*n* = 465), in-hospital cardiac arrest (*n* = 37), pediatric SCAs (*n* = 61), patient transfer to another hospital (*n* = 38), and blood type unknown or missing from records (*n* = 1,521). The remaining 2,601 patients were included in our analysis. Among these, 16.5% (*n* = 429) survived to hospital discharge, and 10.7% (*n* = 278) had good neurological outcomes at hospital discharge.

### 3.2 Comparison of variables between survival and neurological outcome groups

Table 1 summarizes the baseline characteristics of the study population. The mean age of the cohort was 70.65 ± 15.42 years, and 62.6% were male. The mean BMI was 23.06 ± 5.02. The proportions of ABO blood types in the survival group did not differ significantly from those in the mortality group, with blood type O accounting for 41.5% versus 41.0%, A for 28.0% versus 28.5%, B for 24.2% versus 25.7%, and AB for 6.3% versus 4.8%, respectively. There was also no significant difference in blood type distribution between the good and poor neurological outcome groups, with blood type O in 41.0% versus 41.1%, A in 27.7% versus 28.5%, B in 23.7% versus 25.7%, and AB in 7.6% versus 4.7%, respectively.

**TABLE 1:** Patient information: demographics, pre-arrest diseases, resuscitation events, and laboratory data.

Variables	Total (*n* = 2,601)	Mortality (*n* = 2,172, 83.5%)	Survival (*n* = 429, 16.5%)	*P*	Poor CPC (*n* = 2,323, 89.3%)	Good CPC (*n* = 278, 10.7%)	*P*
Age	70.65 ± 15.42	72.42 ± 14.98	61.67 ± 14.43	< 0.001	72.01 ± 15.02	59.32 ± 13.93	< 0.001
Sex, males	1,629 (62.6)	1,317 (60.6)	312 (72.7)	< 0.001	1409 (60.7)	220 (79.1)	< 0.001
BMI	23.06 ± 5.02	22.94 ± 5.14	23.61 ± 4.40	0.009	22.9 ± 5.11	24.24 ± 4.17	< 0.001
Blood type				0.839			0.526
O	1,068 (41.1)	890 (41.0)	178 (41.5)		954 (41.1)	114 (41.0)	
A	739 (28.4)	619 (28.5)	120 (28.0)		662 (28.5)	77 (27.7)	
B	663 (25.5)	559 (25.7)	104 (24.2)		597 (25.7)	66 (23.7)	
AB	131 (5.0)	104 (4.8)	27 (6.3)		110 (4.7)	21 (7.6)	
**Pre-arrest comorbidities**							
Hypertension	1,193 (45.9)	1,017 (46.8)	176 (41.0)	0.027	1,085 (46.7)	108 (38.8)	0.012
Diabetes mellitus	810 (31.1)	705 (32.5)	105 (24.5)	0.001	753 (32.4)	57 (20.5)	< 0.001
Hyperlipidemia	239 (9.2)	170 (7.8)	69 (16.1)	< 0.001	191 (8.2)	48 (17.3)	< 0.001
Chronic kidney disease	511 (19.6)	449 (20.7)	62 (14.5)	0.001	472 (20.3)	39 (14.0)	0.005
Malignancy	621 (23.9)	524 (24.1)	97 (22.6)	0.502	575 (24.8)	46 (16.5)	0.001
**Resuscitation events**							
Witnessed collapse	1,397 (53.7)	1,073 (49.4)	324 (75.5)	< 0.001	1176 (50.6)	221 (79.5)	< 0.001
Bystander CPR	1,084 (41.7)	840 (38.7)	244 (56.9)	< 0.001	907 (39.0)	177 (63.7)	< 0.001
Response time	15.31 ± 6.30	15.43 ± 6.22	14.73 ± 6.70	0.219	15.38 ± 6.29	14.78 ± 6.38	0.371
Initial shockable rhythm	206 (7.9)	128 (5.9)	78 (18.2)	< 0.001	148 (6.4)	58 (20.9)	< 0.001
Defibrillation	420 (16.1)	323 (14.9)	97 (22.6)	< 0.001	352 (15.2)	68 (24.5)	0.001
Low-flow time	28.49 ± 20.61	29.92 ± 20.88	20.88 ± 17.20	< 0.001	29.4 ± 20.86	20.45 ± 16.21	< 0.001
Etiology				< 0.001			< 0.001
Cardiac	983 (37.8)	684 (31.5)	299 (69.7)		752 (32.4)	231 (83.1)	
Infection/sepsis	346 (13.3)	307 (14.1)	39 (9.1)		333 (14.3)	13 (4.7)	
Others	1,272 (48.9)	1,181 (54.4)	91 (21.2)		1,238 (53.3)	34 (12.2)	
**Laboratory data**							
White blood cell	14.01 ± 19.59	13.91 ± 20.91	14.36 ± 14.10	0.682	13.94 ± 20.03	14.43 ± 16.78	0.707
Neutrophil-seg	51.33 ± 21.1	51.34 ± 21.51	51.31 ± 19.68	0.984	51.39 ± 21.41	50.97 ± 19.28	0.765
C-reactive protein	4.90 ± 7.65	5.52 ± 8.18	2.29 ± 3.97	< 0.001	5.31 ± 7.96	1.37 ± 2.23	< 0.001
Lactic acid	12.48 ± 5.69	13.33 ± 5.63	9.72 ± 4.98	< 0.001	13.02 ± 5.62	9.58 ± 5.20	< 0.001
Platelet	199.20 ± 108.90	189.55 ± 109.60	232.40 ± 99.66	< 0.001	193.39 ± 109.10	233.53 ± 101.25	< 0.001
INR	1.55 ± 2.89	1.66 ± 3.20	1.19 ± 1.51	< 0.001	1.62 ± 3.05	1.21 ± 1.83	0.004

Data are presented as mean ± SD or *n* (%), unless otherwise specified. CPC, cerebral performance category (Good: CPC 1-2; Poor: CPC 3-5); CPR, cardiopulmonary resuscitation; INR, international normalized ratio.

Our comparisons of other variables found that the survival group included a higher proportion of males, more witnessed arrests, and greater incidences of bystander CPR, initial shockable rhythm, and defibrillation than the mortality group. The survival group had lower rates of hypertension, diabetes, and chronic kidney disease; was significantly younger; had shorter low-flow times; and exhibited lower levels of CRP, lactic acid, and INR, obtained on the hospital admission. Table 2 summarizes the relationships between variables and survival outcomes. The statistically significant differences in variables between patients in the good neurological outcome group and the poor neurological outcome group were almost the same as those between the survival and mortality groups. Table 3 summarizes the relationships between variables and neurological outcomes.

**TABLE 2:** The association between blood type, covariates and survival outcome.

Variables	Univariate			Multivariable		
	OR	95% CI	*P*	aOR	95% CI	*P*
Age	0.96	0.95–0.96	< 0.001	0.97	0.93–1.00	0.510
Sex, males	1.73	1.38–2.18	< 0.001	1.44	1.05–1.98	0.025
BMI	1.03	1.01–1.05	0.017	0.95	0.89–1.09	0.306
**Blood type**						
O	–	–	Ref	–	–	Ref
A	0.97	0.75–1.25	0.809	0.95	0.68–1.34	0.783
B	0.93	0.71–1.21	0.591	0.84	0.59–1.18	0.314
AB	1.30	0.83–2.04	0.259	1.45	0.81–2.61	0.211
**Pre-arrest comorbidities**						
Hypertension	0.79	0.64–0.98	0.028	0.88	0.65–1.19	0.409
Diabetes mellitus	0.67	0.53–0.86	0.001	0.72	0.51–1.01	0.057
Hyperlipidemia	2.26	1.67–3.05	< 0.001	2.20	1.45–3.33	< 0.001
Chronic kidney disease	0.65	0.49–0.87	0.003	0.78	0.52–1.17	0.225
Malignancy	0.92	0.72–1.18	0.501	–	–	–
**Resuscitation events**						
Witnessed collapse	3.77	2.79–5.11	< 0.001	2.77	1.91–4.00	< 0.001
Bystander CPR	1.83	1.41–2.38	< 0.001	1.49	1.10–2.02	0.010
Response time	0.98	0.95–1.01	0.219	–	–	–
Initial shockable rhythm	3.50	2.58–4.75	< 0.001	2.42	1.57–3.75	< 0.001
Defibrillation	1.67	1.30–2.16	< 0.001	1.49	0.87–1.73	0.198
Low-flow time	0.97	0.97–0.98	< 0.001	0.98	0.96–1.02	0.212
**Etiology**						
Cardiac	–	–	Ref	–	–	Ref
Infection/sepsis	0.29	0.20–0.42	< 0.001	0.29	0.17–0.48	< 0.001
Others	0.18	0.14–0.23	< 0.001	0.22	0.16–0.31	< 0.001
**Laboratory data**						
White blood cell	1.00	1.00–1.01	0.684	–	–	–
Neutrophil-Seg	1.00	1.00–1.01	0.984	–	–	–
C-Reactive Protein	0.91	0.85–0.97	0.005	0.93	0.87–1.05	0.184
Lactic acid	0.87	0.85–0.90	< 0.001	0.95	0.86–1.06	0.245
Platelet	1.00	1.00–1.01	< 0.001	1.00	0.99–1.01	0.739
INR	0.49	0.35–0.69	< 0.001	0.17	0.20–2.00	0.132

Odds ratios (ORs) and adjusted odds ratios (aORs) were obtained from logistic regression models controlling for the following baseline factors: sex, age, comorbidities (hypertension, diabetes mellitus, chronic kidney disease), witnessed arrest, bystander CPR, initial shockable rhythm, lowflow time, and laboratory values (as significant). aOR, adjusted odds ratio; CI, confidence interval; CPC, cerebral performance category (Good: CPC 1-2; Poor: CPC 3-5); CPR, cardiopulmonary resuscitation; INR, international normalized ratio; OR, odds ratio.

**TABLE 3:** The association between blood type, covariates and neurological outcome.

Variables	Univariate			Multivariable		
	OR	95% CI	*P*	aOR	95% CI	*P*
Age	0.95	0.94–0.96	< 0.001	0.97	0.93–1.01	0.209
Sex, males	2.46	1.82–3.33	< 0.001	2.19	1.46–3.29	< 0.001
BMI	1.05	1.03–1.08	< 0.001	0.92	0.81–1.04	0.182
**Blood type**						
O	–	–	Ref	–	–	Ref
A	0.97	0.72–1.32	0.863	1.15	0.77–1.71	0.490
B	0.93	0.67–1.28	0.634	0.84	0.56–1.26	0.407
AB	1.60	0.96–2.65	0.069	1.98	1.02–3.83	0.042
**Pre-arrest comorbidities**						
Hypertension	0.73	0.56–0.94	0.013	0.84	0.59–1.19	0.319
Diabetes mellitus	0.54	0.40–0.73	< 0.001	0.57	0.38–0.85	0.006
Hyperlipidemia	2.33	1.65–3.29	< 0.001	2.19	1.37–3.50	0.001
Chronic kidney disease	0.64	0.45–0.91	0.013	1.14	0.71–1.84	0.578
Malignancy	0.60	0.43–0.84	0.003	0.84	0.55–1.30	0.444
**Resuscitation events**						
Witnessed collapse	3.96	2.72–5.78	< 0.001	2.38	1.54–3.66	< 0.001
Bystander CPR	2.05	1.50–2.82	< 0.001	1.70	1.18–2.44	0.004
Response time	0.98	0.95–1.02	0.371	–	–	–
Initial shockable rhythm	3.67	2.62–5.14	< 0.001	2.16	1.34–3.49	0.002
Defibrillation	1.81	1.35–2.44	< 0.001	1.41	0.86–1.94	0.475
Low-flow time	0.97	0.96–0.98	< 0.001	0.99	0.96–1.02	0.598
**Etiology**						
Cardiac	–	–	Ref	–	–	Ref
Infection/sepsis	0.13	0.07–0.23	< 0.001	0.15	0.07–0.32	< 0.001
Others	0.09	0.06–0.13	< 0.001	0.12	0.08–0.19	< 0.001
**Laboratory data**						
White blood cell	1.00	1.00–1.01	0.709	–	–	–
Neutrophil-seg	1.00	0.99–1.01	0.765	–	–	–
C-reactive protein	0.81	0.70–0.95	0.008	0.79	0.62–1.01	0.063
Lactic acid	0.88	0.85–0.91	< 0.001	0.91	0.80–1.03	0.124
Platelet	1.00	1.00–1.00	< 0.001	1.00	0.99–1.01	0.664
INR	0.59	0.42–0.85	0.004	0.00	0.00–0.85	0.044

Odds ratios (ORs) and adjusted odds ratios (aORs) were obtained from logistic regression models controlling for the following baseline factors: sex, age, comorbidities (hypertension, diabetes mellitus, chronic kidney disease), witnessed arrest, bystander CPR, initial shockable rhythm, lowflow time, and laboratory values (as significant). aOR, adjusted odds ratio; CI, confidence interval; CPC, cerebral performance category (Good: CPC 1-2; Poor: CPC 3-5); CPR, cardiopulmonary resuscitation; INR, international normalized ratio, OR: odds ratio.

### 3.3 Relationship between ABO blood type and survival outcomes in patients with SCA

The odds ratios (ORs) from both the univariate and multivariable analyses showed no differences in the odds of survival for any specific blood type. Univariate analyses comparing the ORs of each other blood type to type O (reference) found that blood type A had an OR of 0.97 (95% CI: 0.75–1.25, *p* = 0.809), blood type B of 0.93 (95% CI: 0.71–1.21, *p* = 0.591), and blood type AB of 1.30 (95% CI: 0.83–2.04, *p* = 0.259). Similarly, in the multivariable analysis, blood type A had an adjusted odds ratio (aOR) of 0.95 (95% CI: 0.68–1.34, *p* = 0.783), blood type B of 0.84 (95% CI: 0.59–1.18, *p* = 0.314), and blood type AB of 1.45 (95% CI: 0.81–2.61, *p* = 0.211).

Among the other variables, factors significantly associated with survival in patients with SCA were male sex (aOR: 1.44, 95% CI: 1.05–1.98, *p* = 0.025), witnesses to cardiac arrest (aOR: 2.77, 95% CI: 1.91–4.00, *p* < 0.001), bystander CPR (aOR: 1.49, 95% CI: 1.10–2.02, *p* = 0.010), initial shockable rhythm (aOR: 2.42, 95% CI: 1.57–3.75, *p* < 0.001), and non-cardiac etiology.

### 3.4 Associations between blood type and good neurological outcomes after SCA

Comparisons to patients with blood type O in the multivariable analysis found that the aOR of good neurological outcomes in those with blood type A was 1.15 (95% CI: 0.77–1.71, *p* = 0.490), and in those with blood type B, it was 0.84 (95% CI: 0.56–1.26, *p* = 0.407). Notably, patients with SCA with blood type AB had a higher aOR of good neurological outcomes, with a statistically significant ratio of 1.98 (95% CI: 1.02–3.83, *p* = 0.042). Other significant factors associated with survival in patients with SCA were male sex (aOR: 2.19, 95% CI: 1.46–3.29, *p* < 0.001), diabetes mellitus (aOR: 0.57, 95% CI: 0.38–0.85, *p* = 0.006), witnesses to cardiac arrest (aOR: 2.38, 95% CI: 1.54–3.66, *p* < 0.001), bystander CPR (aOR: 1.70, 95% CI: 1.18–2.44, *p* = 0.004), initial shockable rhythm (aOR: 2.16, 95% CI: 1.34–3.49, *p* = 0.002), lower INR (aOR: < 0.01, 95% CI: 0.00–0.85, *p* = 0.044), and non-cardiac etiology.

### 3.5 Sensitivity analysis

Table 4 summarizes the results of our sensitivity analyses of three different sub-cohorts. In the sensitivity analysis considering only aseptic SCA etiology, blood type AB had an aOR of 2.21 (95% CI: 1.12–4.35, *p* = 0.023) for good neurological outcomes, but no significant association was noted in survival outcomes. In the analyses restricted to patients with non-cardiac SCA causes, blood type was not significantly associated with either odds of survival or good neurological outcomes. Blood type A showed an aOR of 1.45 (95% CI: 0.82–2.57, *p* = 0.200) for survival and 1.88 (95% CI: 0.81–4.35, *p* = 0.140) for good neurological outcomes. Blood type B had an aOR of 0.91 (95% CI: 0.47–1.76, *p* = 0.776) for survival and 1.18 (95% CI: 0.44–3.12, *p* = 0.745) for good neurological outcomes. Blood type AB had an aOR of 0.94 (95% CI: 0.31–2.83, *p* = 0.911) for survival and 1.41 (95% CI: 0.30–6.65, *p* = 0.663) for good neurological outcomes.

**TABLE 4:** Sensitivity analysis for survival and neurological outcome in three cohorts.

Cohort	Cohort 1: overall population			Cohort 2: non-cardiac origin population			Cohort 3: asepsis population		
Blood type	aOR	95% CI	*P*	aOR	95% CI	*P*	aOR	95% CI	*P*
**Survival outcome**									
O	–	–	Ref	–	–	Ref	–	–	Ref
A	0.95	0.68–1.34	0.783	1.45	0.82–2.57	0.200	0.89	0.61–1.28	0.512
B	0.84	0.59–1.18	0.314	0.91	0.47–1.76	0.776	0.90	0.63–1.28	0.551
AB	1.45	0.81–2.61	0.211	0.94	0.31–2.83	0.911	1.57	0.85–2.92	0.152
**Neurological outcome**									
O	–	–	Ref	–	–	Ref	–	–	Ref
A	1.15	0.77–1.71	0.490	1.88	0.81–4.35	0.140	1.08	0.71–1.62	0.727
B	0.84	0.56–1.26	0.407	1.18	0.44–3.12	0.745	0.89	0.59–1.34	0.563
AB	1.98	1.02–3.83	0.042	1.41	0.30–6.65	0.663	2.21	1.12–4.35	0.023

## 4 Discussion

In this retrospective study of OHCA patients treated at three branches of the NTUH system, the results presented consistent proportions of blood types O, A, B, and AB between those who survived and those who did not, as well as between patients with good and poor neurological outcomes. However, compared to patients with blood type O, those with blood type AB had a nearly two-fold higher likelihood of favorable neurological outcome (aOR: 1.98, 95% CI: 1.02–3.83). Our sensitivity analyses further indicated that in the asepsis cohort, AB type patients showed an approximately 2.21-fold higher likelihood of good neurological outcomes (aOR: 2.21, 95% CI: 1.12–4.35).

### 4.1 Consistency of blood type effects on SCA outcomes and other disease outcomes

The absence of a relationship between blood type and SCA outcomes found in this study is somewhat inconsistent with the general trend in previous literature, which has found non-O blood types to have higher incidences of venous thromboembolism (VTE) and VTE recurrence after discontinuation of anticoagulants, and higher chances of cancer and cancer-related VTE (15, 16, 24, 25). However, it may be that the incidence of VTE and post-SCA neurological outcomes are unrelated. Nonetheless, studies have also reported associations between ABO blood type and various other diseases, including malignancies (13, 14, 26–29). Our study found a significantly lower incidence of malignancy in the good neurological outcomes group (16.5% vs 24.8%, *p* < 0.001). This might explain the more favorable neurological outcomes we saw in blood type AB of the asepsis cohort, as malignancy may have been a mediating factor. However, further research is needed to verify this result. The finding that those with blood type AB had better odds of favorable neurological outcomes in the asepsis SCA cohort was interesting, given that prior studies have shown conflicting results regarding the influence of blood type on the incidence and outcomes of various infectious diseases. Some studies have reported a higher incidence of certain infections with specific blood types, while others have reached opposing conclusions. The finding that blood type AB is associated with better outcomes in the specific context of SCA with distinct sepsis etiology warrants further investigation in future studies.

### 4.2 ABO blood type contribution to SCA outcomes

Our findings contribute to the existing literature by demonstrating a specific association between blood type AB and improved neurological outcomes following SCA. This result is intriguing, as previous studies primarily linked ABO blood type to differences in susceptibility or outcomes in thrombotic diseases, malignancies, and severe infections, rather than neurological recovery post-cardiac arrest (12, 16, 24). Unlike prior studies highlighting blood type O as protective against thrombotic events due to lower von Willebrand factor levels (24), our results identified no survival advantage related to any ABO blood type. Instead, the unexpected neurological benefit seen specifically with type AB, particularly within the aseptic subgroup, suggests that other mechanisms, possibly involving inflammation or microvascular responses to reperfusion, might influence outcomes. Further prospective studies exploring these biological mechanisms are warranted to validate and elucidate the prognostic relevance of ABO blood type in post-cardiac arrest recovery.

ABO blood group does not appear to be a major determinant of survival in patients with SCA. Other prognostic factors were found to play a more substantial role in survival. These included initial cardiac rhythm, low-flow time, whether the arrest was witnessed, and whether bystander CPR was performed. These variables consistently emerged as critical contributors to survival and neurological recovery. Pre-arrest comorbidities such as hypertension, diabetes mellitus, hyperlipidemia, and chronic kidney disease also significantly influence patient outcomes. Moreover, laboratory markers, such as elevated CRP, lactic acid, platelet levels, and INR, are additional prognostic predictors. This diverse range of variables correlated with SCA outcomes demonstrates the importance of comprehensive patient evaluations in the prediction and management of SCA outcomes.

### 4.3 Limitations

This study had several limitations that may have impacted the results and their interpretation. First, the retrospective nature of the study imposed constraints on our ability to establish causation. Retrospective data collection can introduce biases due to the unknown accuracy and stringency of the historical data and its full availability in the records. This may have affected the reliability of the findings. Missing data was a considerable obstacle. Of the 4,723 cases, 1,521 did not have their blood type recorded. Such missing values would have reduced the statistical power of our findings and could have skewed our results. Second, selection bias was a concern due to the study’s reliance on a single medical system—the National Taiwan University Hospital system. This may have limited the representativeness of the sample, as patients treated within this specific system may differ in significant ways from those treated in other institutions or geographic regions. Thus, the generalizability of the findings to broader populations is restricted. Third, race or ethnicity was not included in the final models due to the limited heterogeneity in our patient population and incomplete recording of such data. Future research across more diverse populations is warranted to evaluate racial or ethnic differences. These limitations necessitate caution in the interpretation of our results.

## 5 Conclusion

Our study found no significant relationship between ABO blood type and survival outcomes. However, patients with blood type AB had significantly better odds of a favorable neurological outcome than those with blood type O. This trend persisted when we analyzed only the asepsis cohort.

## Data Availability

The data analyzed in this study is subject to the following licenses/restrictions: the data that support the findings of this study are available on request from the corresponding author. The data are not publicly available. Requests to access these datasets should be directed to C-WS, chihweisung@ntu.edu.tw.
